# A comparison of white matter microstructure and correlates with neuropsychological measures in younger and older adults

**DOI:** 10.1371/journal.pone.0305818

**Published:** 2024-06-24

**Authors:** Abu-Bakar Sheriff, Vanessa Scarapicchia, Erin L. Mazerolle, Brian Christie, Jodie R. Gawryluk

**Affiliations:** 1 Division of Medical Sciences, University of Victoria, Victoria, BC, Canada; 2 Department of Psychology, University of Victoria, Victoria, BC, Canada; 3 Institute on Aging and Lifelong Health, University of Victoria, Victoria, BC, Canada; 4 Department of Psychology, St. Francis Xavier University, Antigonish, NS, Canada; University of Rochester, UNITED STATES

## Abstract

**Objective:**

With a globally aging population, there is a need to better understand how brain structure relates to function in healthy older and younger adults.

**Methods:**

34 healthy participants divided into older (17; Mean = 70.9, SD = 5.4) and younger adults (17; Mean = 28.1, SD = 2.8) underwent diffusion-weighted imaging and neuropsychological assessment, including the California Verbal Learning Test 2nd Edition and the Trail Making Test (TMT-A and TMT-B). Differences in white matter microstructure for older and younger adults and the association between DTI metrics (fractional anisotropy, FA; mean diffusivity, MD) and cognitive performance were analyzed using tract-based spatial statistics (p < 0.05, corrected).

**Results:**

Older adults had significantly lower FA and higher MD than younger adults in widespread brain regions. There was a significant negative correlation between executive function (TMT-B) and MD for older adults in the right superior/anterior corona radiata and the corpus callosum. No significant relationship was detected between DTI metrics and executive function in younger adults or with memory performance in either group.

**Conclusions:**

The findings underscore the need to examine brain-behaviour relationships as a function of age. Future studies should include comprehensive assessments in larger lifespan samples to better understand the aging brain.

## Introduction

There has been a global shift in population demographics, such that the number of individuals over the age of sixty years currently outnumber youth under age five [1]. With an aging population, it is imperative to understand changes in brain structure throughout the course of adulthood, and how these relate to performance on neuropsychological assessment. Many studies have used magnetic resonance imaging (MRI) to examine the aging brain because it is widely accessible and easy to repeat. However, many of these studies have examined differences or changes specific to grey matter volume, and fewer have examined white matter microstructure using techniques such as diffusion tensor imaging (DTI). Studies focused on white matter have demonstrated decreased white matter integrity in older adults (OA) compared to younger adults (YA) [e.g. 2, 3]. Furthermore, although many studies have examined the correlates of DTI metrics and neuropsychological performance in various conditions, such as Alzheimer’s disease and traumatic brain injury, most studies have used experimental tasks rather than neuropsychological assessment measures that are typically used in clinical practice. Relatively few studies have used traditional neuropsychological measures combined with DTI to study healthy OA. Research to date on OA has revealed that reduced microstructural integrity of white matter relates to slower processing speed [4, 5], executive function [6] memory [7] and language [8, 9], although more studies are needed.

The current study had two main objectives: 1) to derive DTI metrics of white matter microstructural integrity and compare groups of healthy OA and YA; and 2) to examine the relationship between DTI metrics and cognitive performance using neuropsychological assessment measures of memory and executive function in both OA and YA.

It was hypothesized that the OA group would have significantly lower white matter integrity than the YA group, particularly in the frontal and temporal lobes. It was also expected that measures of memory and executive function would correlate with DTI metrics in both groups, but to a greater extent in older adults.

## Methods

### Participants

Participants were recruited from the community between June-December 2019, based on age with a younger adult group ranging from age 25–35 years and an older adult group aged 65 years and up. Inclusion criteria specified English speakers, with normal or corrected-to-normal vision. Participants also needed to be “neurologically healthy”, defined as no history of major neurological conditions (e.g. neurodegenerative disorders) or psychiatric disorders (e.g. schizophrenia). Individuals were excluded based on contraindications for MRI. The study protocol was approved by the Human Research Ethics Board at the University of Victoria and all participants provided their written informed consent.

### Study design

The currently presented data were collected as part of a larger multi-modal study with additional non-overlapping findings presented in [10, 11].

### DTI acquisition

Data were collected at West Coast Medical Imaging (Victoria, BC) on a 3T GE Signa Pioneer MRI scanner. The images were acquired with a SE-EPI sequence, axially, with the following parameters: TR = 8000 ms, TE = 101 ms, flip angle = 90°, 52 slices (no gap), voxel size = 1.4 x 1.4 x 2.0 mm. There were 48 images acquired for each scan: 45 diffusion-weighted images (b = 1000 s/mm^2^) and 3 non-diffusion-weighted images (b = 0 s/mm^2^; b0). Each acquisition took approximately 6 minutes.

### Neuropsychological assessment

Memory was measured with the California Verbal Learning Test 2^nd^ Edition (CVLT-II) and executive function with the Trail Making Test (TMT- A and TMT -B). CVLT-II is a word-list learning task with a delayed condition to test recall. TMT-A and TMT-B are visual attention and task-switching tests used to evaluate visuo-motor processing speed and alternating attention. Z-scores were computed for each measure to correct for age, sex, and level of education.

### DTI analyses

Analyses were performed using Functional MRI of the Brain Software Library (FSL) version 5.0.10 [12, 13]. Diffusion weighted images were aligned to the initial b0 image using the eddy correction tool to correct for head motion and distortions. Brain tissue was then differentiated from the skull using the brain extraction tool, with results visually inspected for accuracy. The DTIFit tool was used to create fractional anisotropy (FA) and mean diffusivity (MD) images for the tract-based spatial statistics (TBSS) pipeline (https://fsl.fmrib.ox.ac.uk/fsl/fslwiki/TBSS). TBSS steps included: erosion of the images to remove outliers from DT fitting, nonlinear registration to standard space (FMRIB_FA_58) and derivation of a mean FA skeleton with a threshold of 0.2. Voxel-wise statistics were performed on the skeleton using randomise to compare differences between groups, for both FA and MD. This tool computed an independent sample t-test, using 5000 permutations and correction for multiple comparisons with threshold free cluster enhancement. with threshold-free cluster enhancement. Sex and years of education were included as regressors of no interest, to remove confounding effects from the images as per [14]. Each group was then examined separately within the TBSS pipeline using randomise to evaluate the correlation between domains of cognition (memory and executive function) and white matter microstructure (FA and MD). Age, sex, and years of education were included as regressors of no interest. All analyses were corrected for multiple comparisons (p < 0.05, corrected). Regions of significance were identified using the Johns Hopkins University ICBM DTI-81 white matter atlas.

## Results

### Participants

Participants included 34 healthy adults, including 17 younger and 17 older adults. Demographic characteristics are presented in Table 1.

**Table 1:** Participant demographic characteristics and cognitive performance.

	Younger	Older	*p*-value
	Adults	Adults	
Age (years)	28.1 ± 2.8	70.9 ± 5.4	*p* = 0.0099 × 10^−12^
Age Range	25–35 (10)	65–82 (17)	-
Education (years)	17.7 ± 3.2	17.2 ± 3.0	*p* = 0.6177
Education Range	12–23 (11)	9–23 (14)	-
Number of Males	8	8	-
Number of Females	9	9	-
TMT B (Z score)	0.1034	1.312	*p* = 0.0046
	± 1.1439	±1.1703	
CVLT-II	-0.0588	0.4117	*p* = 0.1930
Long Delay	±1.1843	±0.8521	
Free Recall (Z score)			

### Microstructural white matter differences between healthy older and younger adults

**Fig 1:**
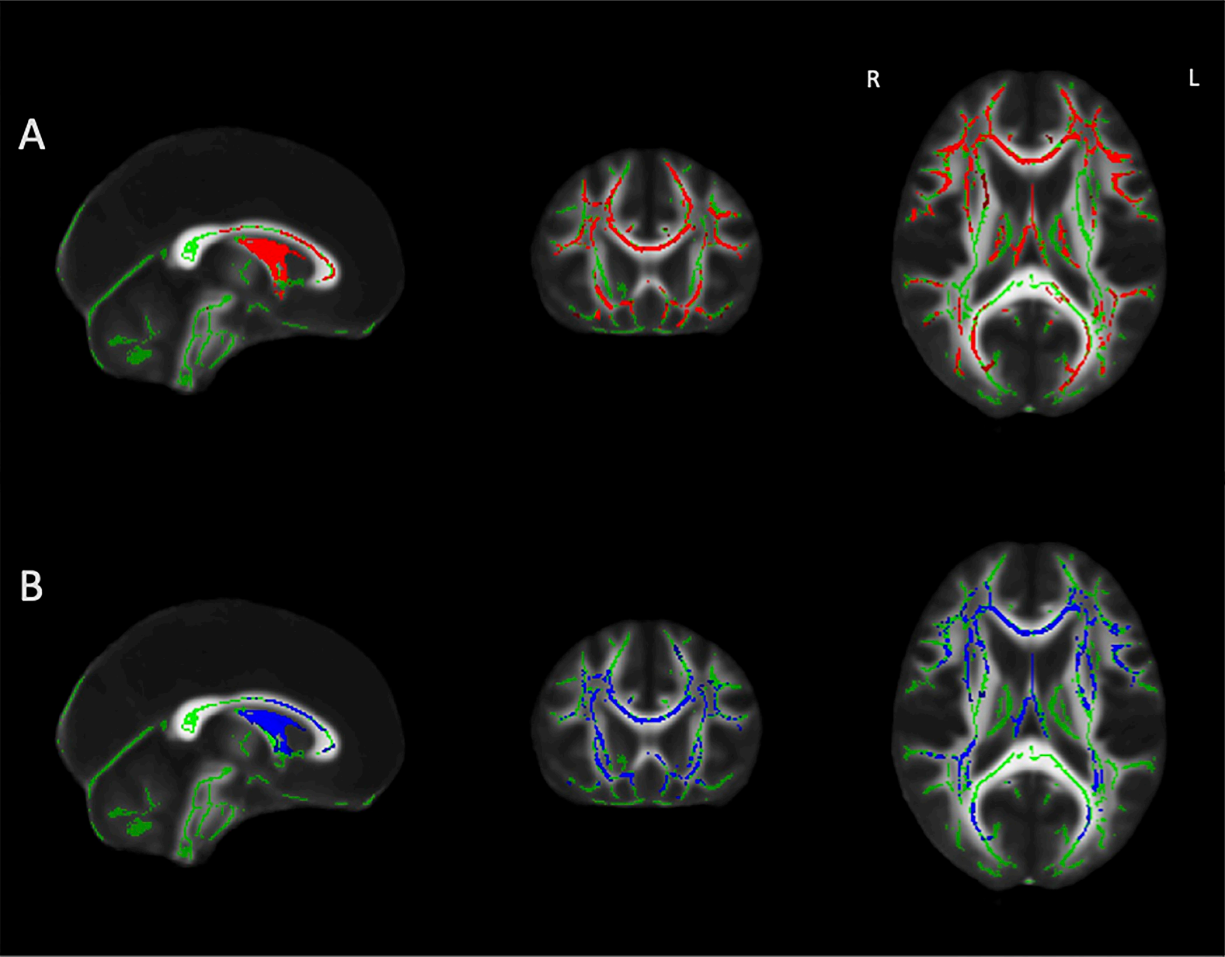
From left to right: sagittal, coronal and axial slices of the standard FMRIB_FA_58 brain displaying results of TBSS analyses overlaid on the white matter skeleton (green) showing regions that A) have significantly lower FA (red) and B) higher MD (blue) in older adults compared to younger adults (p<0.05, corrected for multiple comparisons, sex, and years of education). MNI coordinates: 0, -18, 18.

Comparison of FA and MD values between groups revealed significantly lower FA and higher MD in OA compared to YA in regions including within the left cerebral peduncle, posterior limb of the internal capsule, right anterior limb of the internal capsule, bilateral posterior thalamic radiations, superior longitudinal fasciculi, anterior corona radiata, cingulum, superior coronal radiata, posterior corona radiata, external capsule, optic radiations, stria terminalis, retrolenticular parts of the internal capsule, crura of the fornix, sagittal strata, inferior fronto-occipital fasciculi, inferior longitudinal fasciculi, as well as the body of the fornix and the splenium, genu, and body of the corpus callosum (Fig 1). There were no regions where YA had decreased white matter microstructure compared to OA.

### Relationship between microstructural white matter and cognitive performance

**Fig 2:**
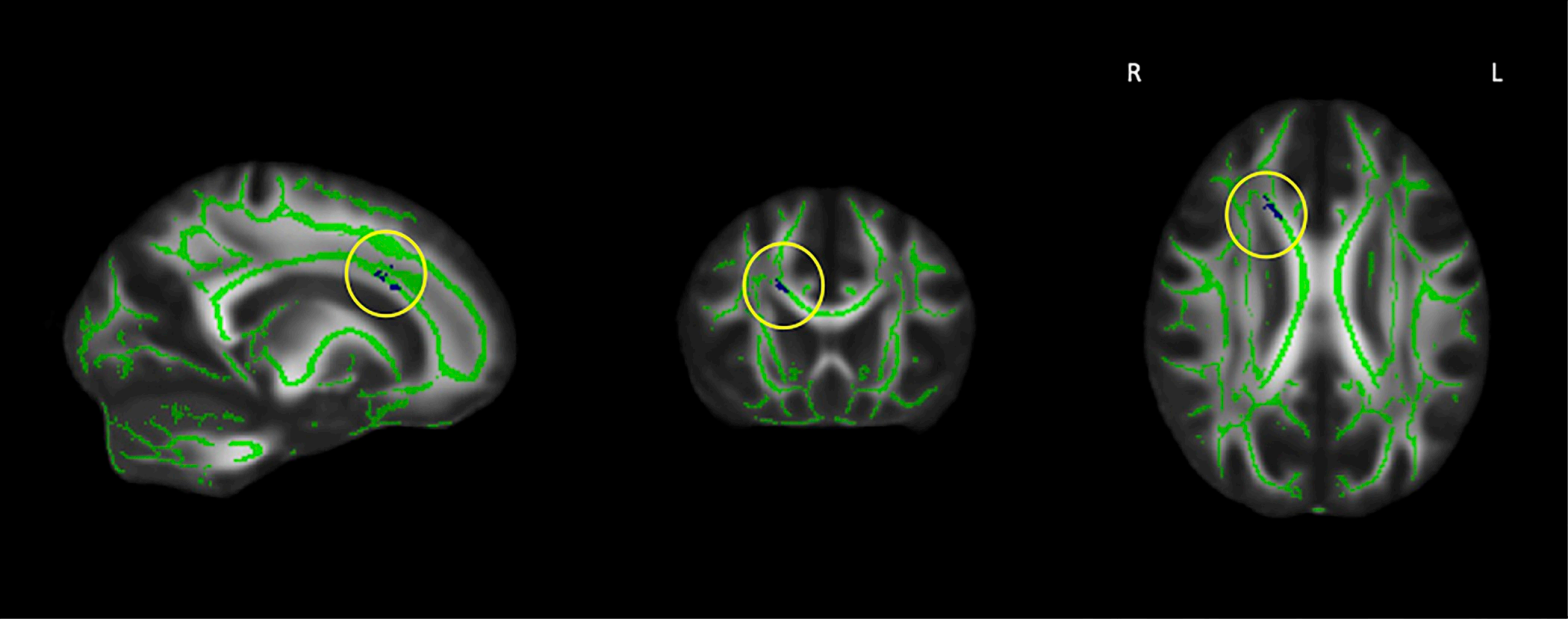
From left to right: sagittal, coronal, and axial slices of the standard FMRIB_FA_58 brain displaying results of TBSS analyses showing regions (circled in yellow) that with a statistically significant negative correlation between MD and executive function (blue) overlaid on the white matter skeleton (green) in older adults (MNI coordinates: 19, 15,32; right superior corona radiata; 18, 19, 27; right anterior corona radiata; 15, 16, 27; body of the corpus callosum) (p < 0.05, corrected for multiple comparisons as well as age, sex, and years of education).

Performance on TMT-B and CVLT-II Long Delay Free Recall are presented for each group in Table 1. Analyses investigating the relationship between DTI metrics and memory performance did not detect significant relationships in healthy OA or YA. With regards to executive function, a significant negative correlation was observed with MD in the OA group in the right superior and anterior corona radiata and the body of the corpus callosum (Fig 2). To examine if these findings could relate to processing speed, a post-hoc examination of TMT-A scores and MD was performed in the OA group; there were no significant findings. No significant correlations were found between executive functioning and FA in either the OA group or the YA group.

## Discussion

The current study aimed to examine differences in white matter microstructure between YA and OA, and relationships between white matter microstructure and cognitive performance on measures of memory and executive function in YA and OA.

The first hypothesis was that OA would show decreased FA and increased MD in widespread regions, including the temporal and frontal lobes, compared to YA. As expected, analyses revealed that OA had significantly lower FA and higher MD than YA in widespread regions. These results are consistent with Ziegler et al., [2], who found decreased FA in older compared to younger participants in diffuse regions using a whole brain approach. These findings are also in line with those of Boekel and Hsieh [3], who found a negative correlation between age and FA across diffuse regions within middle to older aged adults. These cross-sectional studies are also in line with findings from longitudinal studies that have demonstrated decreases in white matter integrity over time in healthy aging adults. For example, Moscufo et al [15] examined older adults at baseline and four years follow up and found decreased white matter integrity over time. Interestingly, Ouyang et al. [16] took a similar approach, but compared middle aged adults (36–64 years) to younger adults (20–35 years) and found widespread differences reflective of reduced white matter microstructure (e.g. decreased FA) in the middle-aged group. It is possible that the observed between group differences in the current study could have been detected prior to age 65 years, although further research is needed that incorporates a lifespan approach including young, middle and older adult groups.

The second hypothesis was that measures of memory and executive function would correlate with DTI metrics in both groups, but to a greater extent in OA. Although executive function refers to a broad array of cognitive abilities, the current study used a measure of alternating attention, TMT-B, as a proxy. The findings revealed that poorer performance on the TMT-B task was associated with increased MD in the OA group, specifically in the right superior and anterior corona radiata as well as the body of the corpus callosum, suggestive of reduced white matter integrity. To investigate whether the findings are explained by the simple visual-motor processing speed component of the task, the relationship between MD and TMT-A scores were also examined for the OA group, with no significant findings. Therefore, it is presumed that the alternating attention component unique to the TMT-B led to the significant finding for the white matter microstructure. Previous literature has established an important role for the frontal lobe in executive function (e.g. [17–20]). The right lateralization of the findings may be due to the visuospatial component of the TMT tasks, for which the right hemisphere is typically more dominant [21]. Although the significant region identified in the current study was small, it is possible that other types of executive function tasks may correlate with other frontal lobe regions. For example, Charlton et al. [22] examined middle and older adults (mean age 69 years) using several working memory tasks, including digit span and letter number sequencing, and found a significant relationship with white matter microstructure in the inferior frontal lobe and left frontal lobe near the corpus callosum, as well as the posterior cingulum and aspects of the temporal lobe. Kantarci et al. [23] also studied older adults (median age 79 years) using the TMT-B in combination with digit span as a proxy of executive function and found relationships with white matter microstructure in the cingulum and inferior longitudinal fasciculus. Macpherson et al. [24] were specifically interested in the relationship between TMT-B performance and brain structure in OA (mean age 76 years). Their findings demonstrated a positive relationship between TMT-B completion time and FA in the uncinate fasciculus and a negative relationship with MD in the anterior thalamic radiation, although these relationships did not remain when complex processing speed (e.g. symbol digit modalities test performance) was included in their model. It is possible that different combinations of measures, and varied age groups have led to disparate findings across studies.

Although a similar finding was expected, albeit to a lesser extent for young adults, there was no significant relationship between executive function performance and DTI metrics. It is possible that floor and ceiling effects of MD and FA, respectively, may have dampened any potential relationships between DTI metrics and cognitive performance in the YA group.

As an index of memory, the long-delay free recall condition of the CVLT-II was used. There was no significant relationship between memory and DTI metrics in either YA or OA. Several studies have examined relationships between measures of memory and white matter microstructure with significant findings, although these have largely focused on individuals with neurological conditions (e.g. [25]) and some have taken a more sensitive region of interest approach (e.g. [26]). Interestingly, Kantarci et al. [23] also examined verbal and visual memory function using a combination of the Wechsler Memory Scale -Revised Logical Memory and Visual Reproduction and the Rey Auditory Verbal Learning Test and found significant relationships with white matter microstructure (FA) in the inferior longitudinal fasciculus and anterior and posterior cingulum. Further studies using comprehensive neuropsychological assessment measures across the lifespan will be important to establish the relationship, if any, between brain structure and memory function across ages.

### Limitations

Although the current study examined both brain structure and cognitive performance in two age groups, there are several limitations to this work that can be addressed in future research. First, the current study used a cross-sectional approach and offers a comparative analysis between different groups of people rather than a longitudinal aging study, which limits the applicability of these results to the actual aging process. Future approaches will use one group of participants and compare their DTI metrics over time.

There are also limitations related to the data acquisition and analyses. Specifically, even though DTI metrics such as FA and MD are commonly used to examine white matter microstructure, the complexity of white matter (e.g., level of myelination, potential crossing or kissing fibers) can lead to limitations in interpretation [27, 28]. Furthermore, the TBSS analysis approach is limited by the accuracy of each step and may be prone to errors based on aspects such as the quality of image registration needed for creation of a mean FA skeleton [29]. The current study carefully screened each step in the processing pipeline to ensure accuracy.

The current study focused on the most commonly examined DTI metrics of FA and MD because these are most comparable with existing literature. It is possible that metrics such as radial diffusivity (RD) and axial diffusivity (AD) could provide additional information related to myelination and axonal degeneration, respectively, in future studies. For example, Bennett et al., [30], examined FA, RD, and AD and found that age related decreases in FA were associated with several patterns of increases and decreases in AD and RD that were thought to reflect different changes in white matter (e.g. in myelination or axonal loss) related to aging.

Relatedly, the current study was specifically interested in how DTI metrics differed as a function of age. As a result, age related variables, such as white matter hyperintensities (WMH), were not included as regressors of no interest. Rather, it is expected that the DTI metrics would broadly reflect white matter alterations related to age. Notably, some other studies have combined younger and older adult groups and specifically examined the effects of WMH and have found relationships with cognitive performance (e.g. [31]). Future studies should further consider the effects of WMH.

Additionally, the participant groups were pooled from a sample with high education, which may indicate some protective effects on white matter integrity. Sampling from a more diverse population, in terms of education and socioeconomic status will lead to the results being more generalizable to the public at large.

## Conclusions

The current study focused on white matter microstructure and examined the relationships between structural integrity and performance on traditional neuropsychological assessment measures. The findings revealed widespread reduced white matter integrity in OA compared to YA. There were significant correlations between performance on TMT-B and a metric of white matter integrity. Future research should expand the current findings with larger sample sizes and more comprehensive assessment approaches. Understanding the structural brain changes expected in healthy aging, and their relationship with widely used standardized cognitive measures, will help neuropsychologists better identify and differentiate normal cognitive aging from age-related pathology.

## References

[1] World Health Organization. *Ageing and health*. Oct, 2022.

[2] Ziegler D. A., Piguet O., et al. Cognition in healthy aging is related to regional white matter integrity, but not cortical thickness. *Neurobiology of aging*, 2010; 31(11): 1912–1926. doi: 10.1016/j.neurobiolaging.2008.10.015 19091444 PMC2996721

[3] Boekel W., & Hsieh S. Cross-sectional white matter microstructure differences in age and trait mindfulness. *PloS one*, 2018; 13(10): e0205718. doi: 10.1371/journal.pone.0205718 30321218 PMC6188777

[4] Kerchner G. A., Racine C. A., et al. Cognitive processing speed in older adults: relationship with white matter integrity. *PloS one*, 2012; 7(11): e50425. doi: 10.1371/journal.pone.0050425 23185621 PMC3503892

[5] Kuznetsova KA, Maniega SM, Ritchie SJ, Cox SR, Storkey AJ, Starr JM, et al. Brain white matter structure and information processing speed in healthy older age. Brain Struct Funct. 221(6):3223–35. doi: 10.1007/s00429-015-1097-5 26254904 PMC4920858

[6] Ystad M., Hodneland E., et al. Cortico-striatal connectivity and cognition in normal aging: a combined DTI and resting state fMRI study. *NeuroImage*, 2011; 55(1): 24–31. doi: 10.1016/j.neuroimage.2010.11.016 21073962

[7] Shim G., Choi K. Y., Kim D., et al. Predicting neurocognitive function with hippocampal volumes and DTI metrics in patients with Alzheimer’s dementia and mild cognitive impairment. *Brain and behavior*, 2017; 7(9): e00766. doi: 10.1002/brb3.766 28948070 PMC5607539

[8] Houston J, Allendorfer J, Nenert R, Goodman AM, Szaflarski JP. White Matter Language Pathways and Language Performance in Healthy Adults Across Ages. Front Neurosci. 2019; 13:1185 doi: 10.3389/fnins.2019.01185 31736704 PMC6838008

[9] Yeske B, Hou J, Adluru N, Nair VA, Prabhakaran V. Differences in Diffusion Tensor Imaging White Matter Integrity Related to Verbal Fluency Between Young and Old Adults. Front Aging Neurosci.2021; 13:750621. doi: 10.3389/fnagi.2021.750621 34880746 PMC8647802

[10] Scaraphicchia V., MacDonald S., Gawryluk J.R. The relationship between cardiovascular risk and lifestyle activities on hippocampal volumes in normative aging. *Aging Brain*, 2022: 100033. doi: 10.1016/j.nbas.2022.100033 36908897 PMC9999441

[11] Kwan H., Scarapicchia V., Halliday D., MacDonald S., Gawryluk J.R. Functional near infrared spectroscopy activation during an executive function task differs between healthy older and younger adults. *Aging Brain*, 2022; 2: 100029. doi: 10.1016/j.nbas.2022.100029 36908882 PMC9997178

[12] Jenkinson M., Beckmann C. F., et al. FSL. *NeuroImage*, 2012; 62(2): 782–790. 21979382 10.1016/j.neuroimage.2011.09.015

[13] Smith S. M., Jenkinson M., et al. Advances in functional and structural MR image analysis and implementation as FSL. *NeuroImage*, 2004; 23 Suppl 1: S208–S219. doi: 10.1016/j.neuroimage.2004.07.051 15501092

[14] Matijevic S., Ryan L. Tract specificity of age effects on diffusion tensor imaging measures of white matter health. Frontiers in Aging Neuroscience, 2021; 13. doi: 10.3389/fnagi.2021.628865 33790778 PMC8006297

[15] Moscufo N., Wakefield D. B., et al. Longitudinal microstructural changes of cerebral white matter and their association with mobility performance in older persons. *PloS one*, 2018; 13(3): e0194051. doi: 10.1371/journal.pone.0194051 29554115 PMC5858767

[16] Ouyang Y, Cui D, Yuan Z, Liu Z, Jiao Q, Yin T, et al. Analysis of Age-Related White Matter Microstructures Based on Diffusion Tensor Imaging. Front Aging Neurosci.2021; 13:664911. doi: 10.3389/fnagi.2021.664911 34262444 PMC8273390

[17] Fiske A., & Holmboe K. Neural substrates of early executive function development. *Developmental review*: *DR*, 2019; 52: 42–62. 31417205 10.1016/j.dr.2019.100866 PMC6686207

[18] Müller U., & Kerns K. The development of executive function. In Liben L. S., Müller U., & Lerner R. M. (Eds.), *Handbook of child psychology and developmental science*: *Cognitive processes* (pp. 571–623). 2015; John Wiley & Sons, Inc.

[19] Stuss D. T. Functions of the frontal lobes: relation to executive functions. *Journal of the International Neuropsychological Society*: 2011; JINS, 17(5): 759–765. doi: 10.1017/S1355617711000695 21729406

[20] Stuss D. T., & Benson D. F. The Frontal Lobes and Control of Cognition and Memory. In Perecman E. (Ed.), *The Frontal Lobes Revisited* (pp. 141–158) 1987; Psychology Press.

[21] Springer S. P., & Deutsch G. *Left brain*, *right brain* (3^rd^ edition). W H Freeman/Times Books/ Henry Holt & Co. 1989.

[22] Charlton R., Barrick T.R., et al. White matter pathways associated with working memory in normal aging. *Cortex*, 2010; 46 (4): 474–489. doi: 10.1016/j.cortex.2009.07.005 19666169

[23] Kantarci K, Senjem ML, et al. Diffusion tensor imaging and cognitive function in older adults with no dementia. Neurology. 2011 Jul 5;77(1):26–34. doi: 10.1212/WNL.0b013e31822313dc 21593440 PMC3127333

[24] MacPherson SE, Cox SR, et al. Processing speed and the relationship between Trail Making Test-B performance, cortical thinning and white matter microstructure in older adults. Cortex. 2017; Oct;95:92–103. doi: 10.1016/j.cortex.2017.07.021 28865241 PMC5637162

[25] Mayo CD, Garcia-Barrera MA, Mazerolle EL, Ritchie LJ, Fisk JD, Gawryluk JR; et al. Relationship. Between DTI Metrics and Cognitive Function in Alzheimer’s Disease. *Front Aging Neurosci*. 2019. 10.3389/fnagi.2018.00436 PMC6333848 30687081

[26] van Norden AG, de Laat KF, Fick I, van Uden IW, van Oudheusden LJ, Gons RA, et al. Diffusion tensor imaging of the hippocampus and verbal memory performance: the RUN DMC study. Hum Brain Mapp. 2012. doi: 10.1002/hbm.21231 21391278 PMC6870230

[27] Figley CR, Uddin MN, Wong K, Kornelsen J, Puig J, Figley TD. Potential Pitfalls of Using Fractional Anisotropy, Axial Diffusivity, and Radial Diffusivity as Biomarkers of Cerebral White Matter Microstructure. Front Neurosci. 2022. doi: 10.3389/fnins.2021.799576 35095400 PMC8795606

[28] Jones DK, Cercignani M. Twenty-five pitfalls in the analysis of diffusion MRI data. NMR Biomed. 2010 Aug;23(7): 803–20. doi: 10.1002/nbm.1543 20886566

[29] Bach M, Laun FB, Leemans A, Tax CM, Biessels GJ, Stieltjes B, et al. Methodological considerations on tract-based spatial statistics (TBSS). Neuroimage. 2014. doi: 10.1016/j.neuroimage.2014.06.021 24945661

[30] Bennett I.J., Madden D.J., Vaidya C.J., Howard D.V., Howard J.H. Jr. Age-related differences in multiple measures of white matter integrity: A diffusion tensor imaging study of healthy aging. Hum Brain Mapp. 2010; Mar;31(3):378–90 doi: 10.1002/hbm.20872 19662658 PMC2826569

[31] Molloy C.J., Nugent S., Bokde A.L.W. Alterations in Diffusion Measures of White Matter Integrity Associated with Healthy Aging. *The Journals of Gerontology*: *Series A*, 2021; Volume 76, Issue 6: 945–954. doi: 10.1093/gerona/glz289 31830253

